# Surgical Strategies for Renal Transplantation: A Pictorial Essay

**DOI:** 10.3390/jcm13144188

**Published:** 2024-07-17

**Authors:** Dorin Novacescu, Silviu Constantin Latcu, Marius Raica, Flavia Baderca, Cristina-Stefania Dumitru, Liviu Daminescu, Razvan Bardan, Vlad Dema, Alexei Croitor, Talida Georgiana Cut, Alin Adrian Cumpanas

**Affiliations:** 1 Department II of Microscopic Morphology, Victor Babes University of Medicine and Pharmacy Timisoara, E. Murgu Square, No. 2, 300041 Timisoara, Romania; novacescu.dorin@umft.ro (D.N.); marius.raica@umft.ro (M.R.); baderca.flavia@umft.ro (F.B.); cristina-stefania.dumitru@umft.ro (C.-S.D.); 2 Angiogenesis Research Center, Victor Babes University of Medicine and Pharmacy Timisoara, E. Murgu Square, No. 2, 300041 Timisoara, Romania; 3 Doctoral School, Victor Babes University of Medicine and Pharmacy Timisoara, E. Murgu Square, Nr. 2, 300041 Timisoara, Romania; vlad.dema@umft.ro (V.D.); alexei.croitor@umft.ro (A.C.); 4 Department of Urology, “Pius Brinzeu” Timisoara County Emergency Hospital, Liviu Rebreanu Boulevard, Nr. 156, 300723 Timisoara, Romania; daminescu75@gmail.com (L.D.); razvan.bardan@umft.ro (R.B.); cumpanas.alin@umft.ro (A.A.C.); 5 Department XV, Discipline of Urology, Victor Babes University of Medicine and Pharmacy Timisoara, E. Murgu Square, Nr. 2, 300041 Timisoara, Romania; 6 Department XIII, Discipline of Infectious Diseases, Victor Babes University of Medicine and Pharmacy Timisoara, E. Murgu Square, Nr. 2, 300041 Timisoara, Romania; talida.cut@umft.ro; 7 Center for Ethics in Human Genetic Identifications, Victor Babes University of Medicine and Pharmacy Timisoara, E. Murgu Square, Nr. 2, 300041 Timisoara, Romania

**Keywords:** kidney/renal transplantation, donor selection, renal graft retrieval, preservation and backbench dissection, surgical challenges and complications, site of implantation, renal graft revascularization, urinary tract reconstruction, post-transplant care, long-term outcomes

## Abstract

This pictorial essay aims to navigate through the complexities and challenges of renal transplantation (RT), by weaving together visual imagery with clinical insights within a comprehensive illustrative surgical guide. Herein, we provide a detailed visual exploration of the intricate anatomy and surgical processes necessary for both renal graft retrieval from the donor and also for an adequate implantation in the recipient. Regarding graft retrieval, after reviewing the relevant retroperitoneal surgical anatomy, and donor nephrectomy techniques, graft preservation and optimal backbench graft dissection principles were meticulously analyzed. Thereafter, the recipient surgical strategy for graft implantation was addressed, focusing on preoperative preparations, the site of implantation selection, exposure, operative bed dissection, graft revascularization, and urinary tract reconstruction. Careful donor and recipient selection, meticulous surgical execution, and rigorous postoperative management clearly hold a pivotal role in optimizing patient outcomes. Fostering a deeper understanding of the surgical nuances and clinical management practices that contribute to successful results post-RT, we hope to provide a useful practical tool for clinicians about to embark on the treacherous road of RT surgery. Innovative technologies and surgical practices that have already significantly improved the safety and effectiveness of RT stand testament to the importance of further scientific inquiry, conceptual developments, and clinical integration. Moving forward, it is essential that the medical community continues to refine these strategies and advocate for equitable access to transplantation, ensuring that advancements in the field translate into real-world benefits for all patients grappling with ESRD. The collaborative efforts of multidisciplinary teams are essential in addressing the complex clinical challenges associated with RT, with the ultimate goal of improving patient survival, enhancing graft longevity, and reducing healthcare disparities.

## 1. Introduction

Renal transplantation (RT) stands as a pivotal therapeutic approach in the management of patients with end-stage renal disease (ESRD) [1]. Thus far, RT clinical management has undergone impactful improvements since its first successful surgical execution over 60 years ago [2]. In fact, with advancements in immunosuppressive medicine, RT now serves as the gold standard treatment for ESRD [3], offering significant cost-effectiveness, quality of life, and survival advantages, compared to long-term dialysis. Conversely, due to further recent progress, modern intensive care units have become quite sophisticated, and perioperative anesthetic techniques have greatly evolved, thus allowing for increasingly complicated recipients to be deemed suitable for RT, while also leading to the expansion of current donor pools by the inclusion of more “marginal donors”, with additional associated complexities [4], and even grafts with congenital anomalies or damaged vessels [2]. Thus, in these situations, the operative risk is greatly elevated.

However, despite these considerable clinical advancements, the RT surgical technique itself, albeit complex, has remained relatively unaltered over time [2]. This interdisciplinary, multistage, major surgical intervention typically involves donor renal graft retrieval and proper preparation, followed by recipient retroperitoneal iliac implantation, i.e., anastomoses for adequate vascular reperfusion and urinary pathway reconstruction. Thus, implicitly warranting focus on particular inherent anatomical/surgical and anesthetic considerations, contemporary RT surgery is usually performed by a specialized interdisciplinary medical team, led by a dedicated transplant surgeon, marking a shift from earlier practices, when urologists and/or vascular surgeons predominantly conducted the procedure [5]. The current study aims to provide a comprehensive pictorial essay on RT surgery, elucidating its anatomical and technical complexities.

Typically, RT recipient candidates are either patients with ESRD, i.e., a glomerular filtration rate (GFR) <15 mL/min/1.73 m^2^, or those with chronic kidney disease (CKD) stage 4 (GFR = 15–29 mL/min/1.73 m^2^), displaying signs of disease progression [6]. However, various contraindications exist:

1. Absolute contraindications: the inability to tolerate surgery due to severe cardiac/pulmonary disease, untreated malignancies, active infections, untreated HIV/AIDS, active drug abuse, uncontrolled psychiatric disease, any medical conditions with a life expectancy of less <2 years, and malignant melanoma within the previous 5 years;
2. Relative contraindications: morbid obesity—recommended body mass index (BMI) <40 kg/m^2^, history of non-compliance with dialysis schedule or medication regimen, frailty, psychiatric issues, cardiovascular/metabolic comorbidities, hepatitis B/C virus infection, previous malignancies—depending on the type, and a limited life expectancy (defined as less than the anticipated waiting time for a kidney) [6,7,8].

Nowadays, in defiance of these variable relative contraindications, RT surgery is often performed on elderly recipients with a variety of preexisting conditions—i.e., diabetes mellitus, cardiovascular diseases—that greatly elevate operative risks. Moreover, impaired platelet function is common, often resulting from a combination of uremia and antiplatelet therapies (e.g., aspirin, clopidogrel). In some instances, recipients may also be on warfarin due to prior thromboembolic diseases or prosthetic heart valves. This multifaceted medical background presents additional challenges in surgical planning and anesthetic management [9].

The survival benefit of kidney transplantation over long-term dialysis is well-documented [10]. Furthermore, the length of time a patient spends on dialysis serves as an independent risk factor for poorer outcomes post-transplantation. In the absence of contraindications, a nephrologist will typically refer the patient to a transplant center, while concurrently initiating preparations for possible dialysis. Even though, for the time being, most renal graft recipients are already on dialysis at the time of RT, some patients may receive a kidney transplant preemptively, sidestepping the need for dialysis altogether [9]. Preemptive RT—conducted before dialysis initiation—is considered optimal, especially for children [11], yet offers only a modest benefit over transplantation following dialysis initiation, which is further nullified if the duration of dialysis before transplantation is less than one year [9]. Furthermore, there is suggestive evidence that patients receiving preemptive transplants may exhibit a lower adherence to immunosuppression therapies [12,13,14]. However, a longer duration on dialysis before RT is independently associated with poorer outcomes [10]. Nevertheless, there are pressing concerns regarding the equity of access to preemptive transplants, i.e., less educated individuals, from lower socio-economic backgrounds, are clearly disadvantaged [9], further emphasizing the need for homogeneous access across different patient demographics.

Simultaneously, in an effort to address the burning need for the increased availability of more viable renal grafts, the RT donor pool has also been greatly diversified recently, with kidneys now being harvested from both living and deceased donors. In fact, nowadays, about 30% of all kidney grafts are retrieved from living-donors, either related or unrelated, usually via laparoscopic donor nephrectomy [15]. Deceased donors typically fall into two categories: either Donation after Brainstem Death (DBD), i.e., donors meet the criteria for formal brain death, or Donation after Circulatory Death (DCD), i.e., the donors are not formally brain-dead, but are deemed unlikely to experience significant neurologic recovery [16]. The procurement process for DCD donors only begins post-cardiac arrest and after a formal declaration by an independent physician. Overall, post-transplantation survival outcomes are generally encouraging. One-year survival rates for DBD transplant recipients are ~97%, and for living donor transplant recipients, ~99% [6].

This pictorial essay aims to navigate through the complexities and challenges of RT surgery by offering a comprehensive illustrative surgical guide. By weaving together visual imagery with clinical insights, we seek to offer an invaluable resource for practitioners, navigating the intricacies of this transformative surgical procedure.

## 2. Renal Allograft Retrieval, Preservation, and Surgical Preparation

Fundamentally, at the core of RT is the critical need to maintain the graft’s viability during the interim from its retrieval to implantation, which is crucial for both immediate and long-term graft function. Thus, the successful transplantation of renal grafts hinges on meticulous preparation, encompassing both the donor and recipient surgical processes. Typically, RT involves two separate dedicated surgical teams: one team to prepare the donor and perform the renal graft retrieval, while another manages the recipient and prepares the operative bed for implantation, either concurrently or subsequently, depending on the type of donor and the logistics required to transport the graft. Particularly in regions like the United States and the Eurozone, where kidneys may travel long distances, between hospitals and/or across state borders, as guided by specific compatibility factors such as Human Leukocyte Antigen (HLA) matching, every effort must be made to further enhance graft longevity. Advanced preservation solutions and automatic machine perfusion systems are employed to mitigate the risks during this critical period [2].

Central to the transplantation process is the surgical removal of a kidney from the donor, known as donor nephrectomy, a procedure that has undergone significant advancements to optimize both the safety of the donor and the efficacy of the transplant. The intricacies of donor nephrectomy, whether from living or deceased donors, necessitate a multifaceted approach tailored to the specifics of each donor and recipient scenario. The surgical approach to donor nephrectomy has been refined over the years, with minimally invasive techniques such as laparoscopic nephrectomy becoming the standard in living donors, due to their benefits in reducing donor morbidity. Laparoscopic nephrectomy is typically favored over traditional open surgery, because it tends to result in less postoperative pain, shorter hospital stays, and a faster return to normal activities for the donor [17].

Donor selection is a comprehensive and critical step that ensures the safety and viability of the transplantation. Candidates undergo a rigorous evaluation that involves a thorough medical examination, psychological evaluation, and various diagnostic tests to assess their suitability. The evaluation aims to confirm the donor’s good health, the functional integrity of their kidneys, and compatibility with the recipient.

### 2.1. Surgical Anatomy Considerations

**Figure 1:**
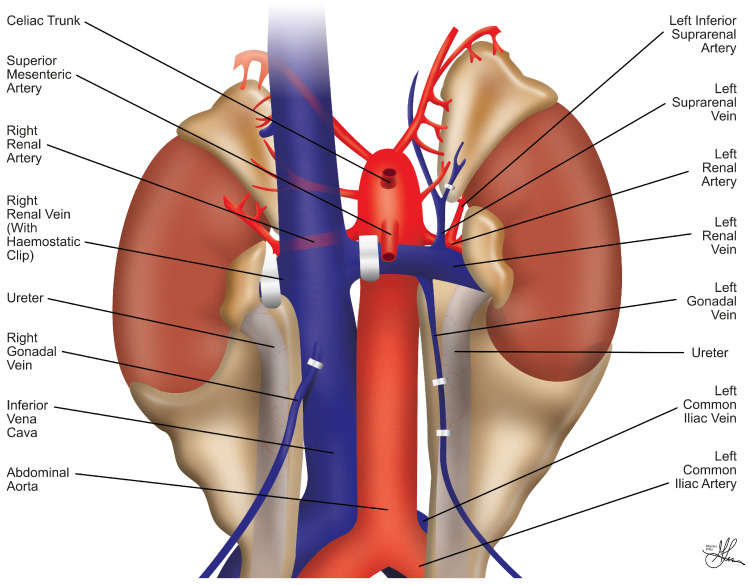
Bilateral renal anatomy in donor, anterior view.

The kidneys represent paired retroperitoneal structures, i.e., situated behind the peritoneal cavity, bilaterally, and they are essential organs for blood filtration. They are abutted superiorly and posteriorly by the diaphragm, and the 11th/12th rib, respectively [18]. Both kidneys rest on the psoas muscle, infero-posteriorly and medially. Each kidney’s upper pole is in close anatomical relation to the adrenal glands [18] (see Figure 1). Enveloping both kidneys is the perinephric fascia, commonly referred to as Gerota’s fascia [19]. The right kidney is neighbored on its anterior borders by the following: the liver antero-superiorly and intimately, through the hepatorenal ligament; the right colonic flexure, latero-superficially, along the Toldt coalescence fascia; and, lastly, the descending part of the duodenum (DII segment), in continuation medially with the head of the pancreas, arranged along the internal renal concavity, i.e., superficially to the renal hilum—namely, the renal vein, possibly the proximal gonadal vein—and more medially, over the inferior vena cava (IVC). In contrast, on the left side, on its anterior aspect, the kidney is flanked by the left colonic flexure, laterally and superficially, similar to the previously described contralateral disposition; the splenic vessels, antero-superiorly; and the lateral body and tail of the pancreas, supero-medially, sometimes in proximity to the renal hilum. Additionally, the spleen is situated antero-medially to the left kidney and is connected anatomically to its upper pole through the lienorenal ligament [20,21].

The renal hilum—where the renal vessels and ureter exit and enter the kidney’s internal concavity, on its medial aspect—displays a recurrent sequential anatomical architecture, with a stable anterior-to-posterior arrangement of structures, from superficial to profound, as follows: the renal vein, then renal artery, and finally the renal pelvis, continued by the ureter [19]. The renal arteries arise laterally from the aorta, just below the origin point of the superior mesenteric artery, and provide nourishment to the kidneys. Notably, the right renal artery runs behind the IVC, before entering the right renal hilum, underneath the ipsilateral renal vein (see Figure 1). The venous anatomy of the kidney mirrors its arterial counterpart, starting from a network of venous capillaries that merge to ultimately form the renal vein, which is generally positioned anterior to the renal artery (see Figure 1). The right renal vein, due to its close proximity to the vena cava, is typically shorter and drains directly into the IVC without any tributaries. Conversely, the left renal vein takes a longer course, crossing anteriorly to the abdominal aorta to drain into the IVC, and typically receives multiple tributaries along the way, including the gonadal, adrenal, inferior phrenic, lumbar, and paravertebral veins [18]. Additionally, the superior mesenteric artery can usually be identified above and in front of the left renal vein, as it traverses the aorta anteriorly (see Figure 1). When it comes to living donations, the left kidney is generally preferred due to its longer renal vein, which facilitates venous anastomosis, thus making the whole graft revascularization process somewhat easier to execute [7].

**Figure 2:**
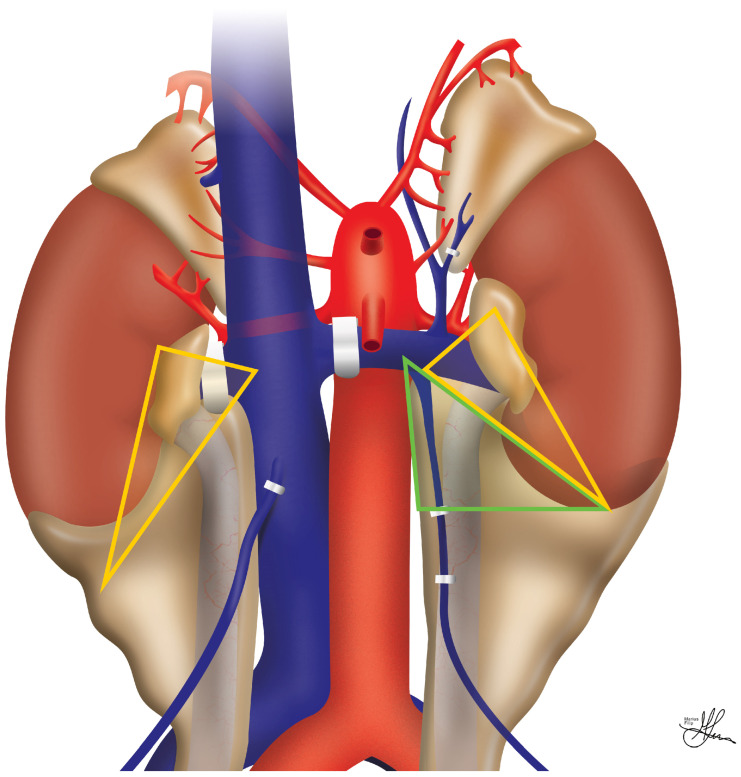
Anatomical borders for the “golden triangle”, highlighted in yellow bilaterally, and the “safety triangle”, highlighted in green on the left side.

Overall, most urological complications post-RT are often linked to technical mistakes made during retrieval, bench dissection, or implantation [22]. Most leaks tend to occur at the distal level of the graft ureter, predominantly at the ureteroneocystostomy (UNCS) site [23]. In lieu of any identifiable technical challenges encountered during urinary tract reconstruction, ischemia and necrosis of the distal ureter, usually due to a surgically compromised blood supply, are considered the primary causes of early ureteral issues post-RT [24]. Unlike native ureters, which receive blood from both renal arteries and pelvic collaterals, the transplanted ureter relies solely on the blood supply from the preserved renal artery branches, near the renal hilum, which run through the peri-ureteric tissues of the graft, particularly within an area referred to as the “golden triangle” (see Figure 2). Thus, this critical region contains vital arterial branches, such as the lower polar artery, which serves the distal ureter but can be easily damaged during retrieval and backbench dissection. The literature strongly emphasizes the importance of preserving these peri-ureteric connective tissues to avoid severe urinary complications [25,26].

Despite a lack of consensus on its specific anatomical boundaries, the “golden triangle” area (see Figure 2) has been generally described by authors as being traditionally contained between the renal hilum, the lower pole of the kidney, and either the right renal vein–IVC junction, or the gonadal–left renal vein junction [25,27,28]. More recent minimally invasive approaches on live donors delineate the “safety triangle”, which is outlined as the space between the lower pole of the kidney and the gonadal vein, extending horizontally until it crosses the gonadal vein, and then following the gonadal vein to its renal vein junction [29], as shown in Figure 2. This even wider safety approach highlights the importance of preserving graft peri-hilar fat vascularity, thereby further minimizing the risk of urological complications postoperatively.

Lastly, detailed anatomical reporting from the donor team to the recipient team is crucial, especially when transporting the graft over distances. This includes the number of arteries, veins, and ureters, and noting any anatomical anomalies or injuries [2]. Such details are vital to prepare the recipient team for the implantation and to prevent any further injury to the graft during backbench dissection.

### 2.2. Donor Nephrectomy Techniques

RT is contingent upon the successful execution of donor nephrectomy, a procedure that continues to be refined across transplant centers worldwide. Practices differ, with some institutions advocating for the anterior transperitoneal approach, through a subcostal, thoraco-abdominal, or even Chevron incision, while others endorse the lumbar extraperitoneal technique, through a posterior flank incision. Notably, the laparoscopic method for renal allograft retrieval from living donors has gained popularity as the preferred standard due to its minimally invasive nature [30].

When executing a donor nephrectomy, it is essential to adhere to several fundamental principles to ensure the integrity and functionality of the donated organ. These principles include the following: (1) achieving proper ample surgical exposure; (2) the delicate manipulation of tissues—particularly during the dissection around the renal artery to prevent vascular spasms; and (3) safeguarding sufficient peri-ureteral and peri-hilar adipose tissue to preserve the vascularity essential for preventing ureteral necrosis post-implantation, i.e., the golden triangle (see Figure 2). Furthermore, the implementation of active diuresis during the procedure is crucial, as it augments the likelihood of immediate graft function post-transplant, an aspect critically important for recipient outcomes [31].

In living donors, the debate between open versus laparoscopic nephrectomy pivots on several factors, including the availability of surgical expertise in laparoscopic methods, the resources necessary to support such techniques, and the specifics of the donor’s prior surgical history, which may impede the feasibility of a laparoscopic approach, i.e., prior abdominal surgeries that may render the laparoscopic method unfeasible. Another consideration is the requisite length of the donor vessels, which may be a determining factor in cases where the vascular anatomy presents marginal challenges. In the postoperative phase, living donors receive meticulous care to manage pain, monitor for signs of complications, and support recovery. The recovery regimen focuses on pain control, early mobilization, and wound care management. Additionally, long-term monitoring of the donor’s renal function and overall health is paramount to ensure their wellbeing. The risk profile for donor nephrectomy is relatively minimal but includes potential for bleeding, infection, and in rare cases, a decrease in the remaining kidney’s function. However, with current surgical techniques and perioperative care, donors typically experience positive outcomes and maintain a normal life span and health quality, which is a testament to the procedure’s safety and effectiveness [32].

In the realm of deceased donors, the predominant procurement technique involves organ retrieval from brain-dead individuals who are physiologically supported by artificial means, termed heart-beating deceased donors, i.e., DBD. The use of such donors often necessitates substantial intravenous (IV) fluid administration to counteract the effects of prior failed interventions, aimed at reducing cerebral edema. To facilitate organ preservation during nephrectomy, diuretics and vasopressors are employed to promote diuresis [33]. Moreover, the administration of systemic heparin and vasoactive agents may be employed to counteract renal vasospasm, as well as to avoid excessive surgical manipulation and the compression of graft vascularity [34]. Conversely, organ donation following cardiac death—non-heart-beating donation or DCD—poses distinct challenges, as it necessitates a rapid organ recovery process to curtail ischemic injury. To mitigate the warm ischemic time, ideally limiting it to under half an hour, swift cooling and an expedited nephrectomy are crucial [3].

Despite the prevalence of brain death (i.e., potential donors) within emergency departments and intensive care settings, numerous patients pass away without this being formally recognized. The potential to harvest suitable grafts from these individuals hinges upon controlling the ischemic damage imminent post-mortem. Strategies like in situ renal flushing and core body cooling via femoral artery and peritoneal catheters placed immediately following cardiac arrest are employed, enabling the prompt transportation of the non-heart-beating donor to the operating room for bilateral nephrectomy [35].

When only the kidneys are to be retrieved from a deceased donor, a generous midline incision allows for en bloc bilateral nephrectomy, with the goal of preserving the full length of the renal vessels, preferably including an aortic Carell patch and vena cava cuff [36]. This technique mitigates the risk of injury to the accessory vessels, present in a small but significant fraction of the population (12–15%), and facilitates the early in situ cooling of the kidneys, reducing the time needed for the procedure [37].

For deceased donors designated for multi-organ retrieval, a meticulous synchronization between surgical teams is imperative to prevent the compromise of any transplantable organ. Anesthesiologists play a pivotal role in preserving the cardiovascular stability of the donor during the extensive dissection required for organ isolation. With organs typically being removed in a sequential manner—heart, lungs, liver, then kidneys—the coordination of these procedures is paramount to maintaining the viability of each organ for transplantation [38].

### 2.3. Allograft Cold Perfusion

From a surgical standpoint, it is imperative to minimize the ischemic time, while also maintaining the graft’s temperature between 1 and 4 °C throughout ischemic manipulation, to reduce potential tissue damage. Simple hypothermia alone does not suffice for maintaining viability. Mandatorily, donor blood must be thoroughly evacuated from the graft and replaced with a suitable preservation solution, in a process called cold perfusion. This practice cools the organ internally, reducing metabolic demand and further protecting against ischemic injury [2].

In deceased donors, graft cold perfusion is achieved during the organ-harvesting process, through direct vascular irrigation, with the preferred pre-prepared preservation solution—commonly, University of Wisconsin (UW) solution, Histidine–Tryptophan–Ketoglutarate (HTK), or other more specialized formulas—being infused directly into the clamped aorta or renal arteries, until complete blood evacuation is achieved [39]. These solutions are selected based on their specific benefits; for example, UW solution extends preservation up to 48 h, allowing ample time for transport and recipient preparation [2].

In living donors, cold perfusion is achieved only after the renal graft had been completely removed from the donor’s body, under hypothermic conditions, i.e., with the graft immersed in an ice-cold basin, using simpler solutions such as lactated Ringer. The effectiveness of these simpler solutions is comparable to more complex ones when the total ischemic time is kept <60 min [40]. Lidocaine, sodium bicarbonate, and heparin have also been reportedly added to enhance the preservation efficacy, along with intra-operative diuretics, like Mannitol or Furosemide, being administered to the donor just prior to arterial clamping to optimize diuresis, minimize renal cell swelling and reduce waste products, thus optimizing the organ’s condition prior to nephrectomy. Subsequently, on the backbench, to achieve the cold perfusion of the living donor renal allografts, a careful cannulation is performed using atraumatic olive-headed heparin needles, to avoid damaging the intima of the vessels [2]. If the primary artery is not readily visible, perfusion may begin via the more accessible renal vein until the artery can be located and prepared. Ensuring complete irrigation before proceeding with delicate dissections is critical to maintaining the integrity of the renal parenchyma, which should exhibit a healthy yellow-pink color, indicating proper perfusion and minimal damage.

Traditionally, ice slush has been used to keep the graft cool during surgery, but this method has limitations, particularly in the robotic-assisted approach. To address these issues, a specialized novel cold ischemia device (CID), designed to maintain a constant and homogeneous low graft temperature during both open and robotic-assisted RT, has been developed [41]. The CID consists of two layers of thin film sealed to create channels for cooling solution flow, with an additional outer insulating layer. The device includes a window to access the renal hilum for vascular anastomosis. In ex vivo tests, the CID was able to maintain a graft temperature <20 °C throughout the simulated RT procedure, outperforming classic ice slush methods [41]. In vivo testing in a porcine model demonstrated the device’s ability to maintain low graft temperatures in both open and robotic-assisted procedures, with mean temperatures at 50 min of 10.8 °C and 14.9 °C, respectively [41].

The CID addresses several limitations of traditional ice slush cooling:

1. More consistent cooling: The device provides the continuous, homogeneous cooling of the entire graft surface.
2. Reduced risk of local/systemic hypothermia: The insulating layer prevents the cooling of the surrounding abdominal organs.
3. Improved surgical access: The device’s design allows for easy access to the renal hilum without compromising cooling.
4. Potential for extended rewarming times: The consistent cooling may allow for longer vascular anastomosis times, particularly beneficial during the learning curve for the robotic approach [41].

Other initiatives have also explored specialized cooling systems for RT. Notably, a silicone renal jacket continuously perfused with methylene blue and ethanol at 4 °C has thus far also shown promising results in a porcine model [42]. This system demonstrated less parenchymal heterogeneity and limited ischemia–reperfusion injuries compared to conventional cooling methods. The development of these devices is particularly important for robotics, where traditional ice slush cooling can be challenging to maintain and may increase the risk of complications such as paralytic ileus [43,44]. By providing more consistent and controlled cooling, these devices may help to expand the indications for robotics and improve reproducibility, especially during the learning curve [41]. While these devices show promise, further research is needed to fully evaluate their impact on clinical outcomes and to assess the safety and efficacy of the CID in larger patient cohorts, including cases with longer rewarming times and specific populations such as deceased donors and grafts with multiple vessels [41,45].

### 2.4. Backbench Allograft Dissection

Backbench preparation of the renal allograft is a crucial step in the transplantation process, performed either before or alongside the recipient’s surgical exposure. If there are concerns about trauma, surgical damage, or the abnormal anatomy of the graft, the surgeon must examine the organ before the recipient’s preparation for surgery, to ensure the graft’s viability and functionality [46]. Additionally, early graft preparation is vital for detecting occult anomalies, like unrecognized tumors or vascular injury, that might preclude transplantation or require complex repairs [9]. Upon removal from cold storage, the kidney undergoes examination, with varying degrees of dissection needed based on whether it is from a deceased or living donor. For kidneys from deceased donors, significant dissection is necessary. All dissections should be conducted delicately, on a proper back table, under adequate lighting, with the kidney immersed in ice slush to maintain its low temperature. The use of microvascular or atraumatic instruments is recommended, to minimize additional trauma to the vessel walls or intima [2,5].

The initial focus is on the renal vein, which is carefully dissected towards the kidney. Small tributaries, as well as the gonadal and adrenal veins on the left side, are managed with clips or silk ties. Small accessory renal veins can be safely ligated due to collateral intercommunication [9]. If two main renal veins of similar size are present, both are preserved to mitigate the risk of venous thrombosis and infarction [5]. For deceased-donor right kidney grafts, a preserved full cylinder of the vena cava is optimal, as it can be modified into a vascular conduit to lengthen the renal vein [5,46,47].

Onward, attention shifts to the renal artery, which is carefully isolated from the surrounding fatty tissue, thus ensuring a clear visualization of the arterial anatomy and preventing injury to any emerging polar arteries. Notably, the right renal artery often extends branches to the suprarenal gland and may also supply the renal upper pole (see Figure 1). In deceased donors, surgeons frequently preserve a Carrel patch of the aortic wall attached to the graft artery. This approach simplifies vascular reconstruction [47], avoiding direct manipulation of the renal artery ostium, and is especially advantageous for pediatric donor kidneys with smaller arterial diameters [5,46].

Multiple arteries are commonly encountered during preparation. While minor accessory arteries supplying the upper pole can often be sacrificed, major accessory vessels, particularly those nourishing the lower pole and critical for ureteral perfusion, must be carefully preserved [2,5,46,47]. Multiple arteries can be consolidated onto a single Carrel aortic patch [48], or kept separate and individually anastomosed to prevent undesirable mechanics on the adjacent graft vein [46]. In cases where atherosclerosis is affecting the donor aorta or renal artery ostium, the artery must be transected at a disease-free level [46]. Lastly, the lymphatics within the allograft are meticulously secured with ties/clips to prevent post-RT lymph leakage and lymphocele formation [49]. It is crucial to maintain adequate peri-ureteral tissue and peri-renal fat around the ureter and lower renal pole (the “golden triangle”—see Figure 2), thus reducing the risk of ureteral ischemia [26].

Upon completion of the dissection, the graft is flushed with 100–200 mL of renal perfusion fluid to eliminate metabolic byproducts and identify any vascular leaks or defects. The renal parenchyma is then assessed, ideally displaying a uniform yellow-pink color, indicative of adequate graft perfusion [46]. Throughout the process, every effort is made to minimize the ischemic time by avoiding unnecessary reconstructions or extended dissections until after at least a partial reperfusion of the graft. The aim is to reduce the total operation duration, thus minimizing potential complications in the recipient. This complex procedure underscores the high level of skill required for the successful backbench preparation of renal allografts, ensuring the best possible outcomes for renal transplantation.

## 3. Preliminary Recipient Surgical Preparations

The established surgical method for first or second single-kidney transplant operations is the open extraperitoneal approach, employed for either iliac fossa [3]. Current evidence does not favor placing a left or right kidney into any specific iliac fossa [50]. Emerging surgical technologies may potentially revolutionize RT by introducing minimal-access approaches. Recently, multiple systematic reviews have compared different graft implantation surgical techniques, including minimally invasive open, laparoscopic, and robot-assisted RT (RART) [51,52]. Specifically, RART offers both advantages and limitations over traditional open RT [52]. The robotic approach is seemingly associated with a lower incidence of delayed graft function (DGF) and fewer postoperative complications, particularly surgical site infections. It also demonstrates similar mid-term functional outcomes, patient survival, and graft survival compared to open RT. These benefits may be especially significant for obese recipients who might otherwise be denied transplantation. However, RART typically requires longer operative and rewarming times, and surgeons face a learning curve of approximately 35 procedures to achieve proficiency. The technique also involves higher upfront costs for equipment and training. Even so, it is important to highlight the current lack of long-term data, with a median follow-up of only 2.2 years in most available studies [52]. Moreover, many of the available studies focus on carefully selected recipients and living donor transplants, potentially limiting the generalizability of the results. Despite these limitations, RART shows promise in potentially reducing complications and expanding access to transplantation.

In fact, particularly in cases where recipients have advanced atherosclerotic vascular disease, RART has faced technical constraints. The main limitation of RART in these atherosclerotic recipients is the lack of haptic feedback, which makes it difficult for surgeons to appropriately locate arterial plaques and safely perform clamping and arteriotomy. To overcome this, a seminal three-dimensional augmented reality (3D AR)-guided approach for RART has been developed [53]. This novel technique involves creating 3D virtual models of the recipient’s common and external iliac arteries, including atherosclerotic plaques, from high-accuracy CT scan imaging. These virtual models, demonstrating high concordance with the actual vessel measurements, are then superimposed onto the real anatomy during the RART procedure using the Da Vinci console software. This allows surgeons to visualize the hidden anatomy of atherosclerotic vessels, guiding them during critical steps of the transplantation. Ultrasound checks showed only minor discrepancies in plaque positioning, with a maximum error of 3 mm. Thus, as this technology continues to evolve and become more refined, it has the potential to make RART accessible to a broader range of patients, including those with advanced atherosclerotic vascular disease [53]. Conversely, the need for randomized studies to validate these findings and determine the long-term safety and efficacy of RART compared to open RT must be emphasized. As the field evolves, RART may play an increasingly important role in RT, but its precise place in the surgical armamentarium remains to be fully established [51,52].

In the case of third or subsequent transplants, pre-operative planning is essential to ensure the right conditions for the renal allograft, i.e., an appropriate arterial inflow, venous outflow, and sufficient space for kidney implantation [54,55]. The nephrectomy of an old transplant kidney may be required before or at the time of transplantation [54]. Mobilization of the common or internal iliac artery, internal iliac vein, or IVC may be necessary [3]. An intra-peritoneal approach, whether through the iliac fossa or midline, may become necessary, albeit rarely [56], and orthotopic transplantation may be warranted in exceptional cases [54,57]. Even so, cohort studies have shown that third or even subsequent renal transplants offer decent patient and graft survival in the short and long term [3].

The approach to nephrectomy in both polycystic kidney disease (PCKD) and failed kidney transplants has evolved towards a more conservative, individualized strategy. Current guidelines no longer recommend a routine pre-transplant nephrectomy for PCKD, with only about a quarter of patients undergoing the procedure overall [58,59]. When necessary, there is a preference for post-transplant and laparoscopic approaches, which are associated with fewer complications and shorter hospital stays [58]. For failed grafts, the decision to perform a nephrectomy also remains controversial [60]. While removing the graft can eliminate a source of inflammation and complications, it risks increasing allosensitization, potentially complicating future transplantation [60,61]. Each possible timing of a graft nephrectomy—pre-transplant, simultaneous with re-transplantation, or post-transplant—carries unique benefits and risks [3].

In both cases, nephrectomy is now generally reserved for specific clinical indications such as recurrent infections, intractable pain, bleeding, or space issues preventing transplantation [58,59]. The trend is moving away from routine nephrectomy solely to create space for a new graft [58,59]. This shift reflects a growing understanding that the potential benefits of nephrectomy must be carefully weighed against the risks for each patient [58,60]. The goal is to optimize outcomes for current and future transplants, while minimizing surgical complications and preserving opportunities for re-transplantation [58,60,61]. As such, decisions regarding nephrectomy should be made on a case-by-case basis, considering the individual patient’s clinical scenario, immunological status, and long-term transplant prospects [58,60].

Orthotopic RT is an alternative surgical technique for kidney transplantation in patients who are unsuitable for standard heterotopic RT in the iliac fossa. The main indications for orthotopic RT include severe atheromatosis of the iliac vessels, occupied iliac fossae from previous transplants, IVC thrombosis, or urinary diversions [62]. The surgical technique, first described by Gil-Vernet et al. in 1978, involves an extraperitoneal approach with end-to-end anastomosis of the graft vessels to the native renal vessels or splenic artery [63]. Various modifications have been described, including the use of the aorta or inferior mesenteric artery for revascularization in cases where the splenic artery is inadequate [62].

The recent literature suggests that orthotopic RT may also be indicated in patients with small renal cell carcinomas in their native kidneys, allowing for simultaneous nephrectomy and transplantation [62,64]. This approach can be particularly beneficial for patients with acquired renal cystic disease, which is common in ESRD and associated with a higher risk of renal cell carcinoma [62]. While orthotopic RT has shown comparable graft and patient survival rates to heterotopic, it is associated with a higher incidence of postoperative complications and reintervention rates [57,62]. The most frequent complications are urinary fistulas or stenosis, occurring at similar rates to heterotopic RT [62]. Despite these challenges, orthotopic RT remains a valuable option for patients unsuitable for heterotopic RT, allowing them to avoid dialysis and its associated complications. However, due to its technical complexity and higher complication rates, it should be reserved for carefully selected patients where standard heterotopic RT is not feasible [57,62].

When listing potential recipients for RT, it is crucial to provide comprehensive information about the associated risks. Patients should be informed of general surgical risks and procedure-specific complications. These include technical issues such as arterial/venous thrombosis, hemorrhage, and urinary complications. The possibility of DGF, which may affect ~50% of deceased donor kidney transplants, should also be discussed. Recipients need to understand the risk of acute rejection and the potential need for biopsies. Clear communication is essential regarding the possible need for biopsy, as well as the requirement for immunosuppressive therapy, which comes with drug-specific side effects and risks associated with immunosuppression. Patient preferences regarding acceptable donors, such as those with preexisting oncological conditions or who are at a high risk for hepatitis or HIV, should be recorded [5].

Immunosuppression typically commences before surgery, regardless of the protocol used. While there is no definitive evidence requiring preoperative immunosuppression, many centers administer a loading dose of a calcineurin inhibitor or an antimetabolite, to achieve effective blood levels immediately post-transplant. Induction agents, commonly basiliximab, are also started before surgery. Recipients of antibody-incompatible grafts typically undergo several days of preoperative immunosuppression and antibody removal [5]. Thus, recipients of organ transplants are particularly susceptible to delayed wound healing and infections due to the immunosuppression required to prevent organ rejection. This necessitates meticulous surgical technique and strict adherence to the basic principles of asepsis and hemostasis [1].

To ensure successful outcomes, it is essential to anticipate, prevent, and promptly address potential surgical complications. One key preventive measure involves securely tying off the lymphatics surrounding the peri-iliac vessels, to mitigate the risk of postoperative lymphocele formation. Careful preparation of the iliac artery and vein segments is necessary to facilitate tension-free vascular anastomoses and optimal positioning of the transplanted organ. Minimizing DGF is a critical concern, and research indicates that reducing anastomosis and/or the rewarming time can be beneficial [65]. This approach may also have positive implications for long-term graft function [66]. The evidence supports the practice of cooling the kidney during implantation [67], which can be achieved through various methods. One technique involves enveloping the organ in a surgical gauze swab filled with crushed frozen saline. An alternative approach utilizes a surgical glove to contain the kidney along with crushed ice, with the vessels externalized through a small incision in the glove. This method not only maintains a low kidney temperature during anastomosis, but also enhances organ handling [5].

### 3.1. Preoperative Considerations

The preoperative work-up for renal transplant recipients requires meticulous attention to numerous factors to ensure the safety and success of the surgery. Upon admission, specific donor-related risks should be discussed with the patient. Informed consent should be obtained. Furthermore, it is essential to conduct a detailed history and physical examination to detect any contraindications for major surgery. Special emphasis should be placed on assessing the patient’s fluid and electrolyte status, as abnormalities such as fluid overload or elevated potassium levels might necessitate preoperative dialysis. The decision to perform dialysis should not be deferred even if it could delay the surgery, especially since patients receiving deceased donor kidneys are at a higher risk of DGF [5].

During the pre-RT evaluation, it is important to assess the arterial and venous inflow through a clinical history and physical examination. A magnetic resonance angiography or computed tomography arteriography should be performed in recipients suspected of having aorto-iliac vascular disease, which could compromise the technical success of RT. For recipients with previously documented deep vein thrombosis (DVT), a duplex echography is recommended to check for residual iliac clots. In rare cases where there is total occlusion of the IVC–iliac system, a portal venous anastomosis might be considered, akin to pancreas transplantation protocols. It is also essential that the patient has a bladder with adequate capacity, appropriate compliance, and a functional continence mechanism before undergoing transplantation. Any necessary bladder rehabilitation or reconstruction should be completed well ahead of the transplant procedure [1].

Prophylactic measures against DVT and pulmonary embolism should include low-dose low-molecular-weight heparin, as per institutional guidelines, thromboembolic deterrent stockings, and perioperative intermittent calf compression. Furthermore, despite being, at least theoretically, a “clean” surgery, the use of prophylactic antibiotics, such as cefuroxime (1.5 g intravenously, at the induction of anesthesia [68]), is still highly recommended, due to the elevated risk of infection, i.e., uremic immuno-compromised recipients [5]. Additional risks include the contamination of the deceased donor kidney during retrieval or a urinary tract infection from a pre-existing urethral catheter. Moreover, implantation involves combined vascular and urinary reconstructions, making the recipient vulnerable to more severe, even catastrophic, complications such as infection at the site of vascular anastomosis, followed by hemorrhage, kidney loss, and distal circulation impairment, thus becoming a life-threatening condition [9]. To further minimize infection risks, thorough skin preparation is essential, including hair removal and disinfecting the abdominal wall with an antimicrobial agent like povidone-iodine or chlorhexidine gluconate [5,9].

### 3.2. Site of Implantation

In primary RT, the right iliac fossa is often preferred because it offers a more horizontal course of the external iliac vein, making venous anastomosis more straightforward. However, each case must be evaluated individually, considering both the recipient’s anatomy and the donor organ’s specific anatomical configuration, particularly the spatial relationships of the artery, vein, and collecting system. When transplanting a right donor kidney into the left side, or vice versa, the collecting system ends up being the most anterior structure, which helps in keeping the ureter away from the iliac vessels, thus enhancing its accessibility. This configuration ensures that the ureter and renal pelvis are positioned antero-medially, which can be advantageous for potential future urinary tract reconstructions, especially if urinary drainage complications arise [3,69].

In obese patients, the right iliac fossa is typically favored for implantation regardless of the donor kidney being used. This preference arises because the iliac vessels are more superficial on the right side, and the right common iliac vein is laterally positioned relative to the common iliac artery, unlike its positioning under the common iliac arterial bifurcation on the left side. For children, placing a kidney into the right iliac fossa or the retroperitoneum is more straightforward, as the common iliac vein and the IVC are readily accessible for venous anastomosis [48].

**Figure 3:**
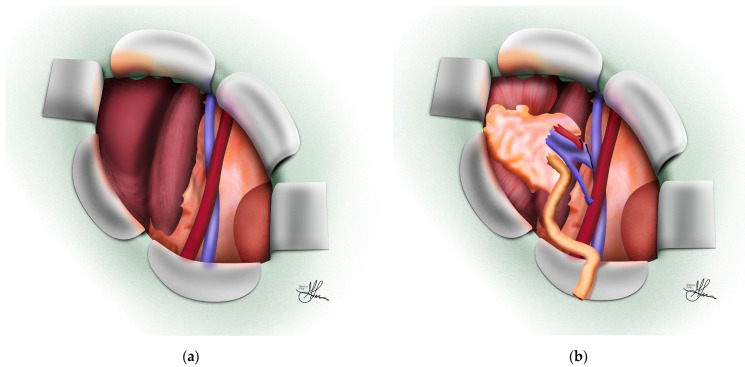
Operative field prepared for graft implantation, oblique view: (**a**) Empty dissected right iliac fossa; (**b**) graft positioned in right iliac fossa.

For diabetic patients receiving a kidney from a living donor who might also be candidates for a subsequent pancreas transplant, the left side is preferred for kidney implantation. This placement allows the right common iliac artery to be used for pancreatic inflow, avoiding ischemia in the kidney from vessel manipulation [5]. In cases where there is a previously failed graft, the opposite iliac fossa is utilized for the second transplant [3]. For a third transplant, the venous connection is typically made to the lower vena cava, and the arterial connection to the lower aorta or common iliac artery on the right, through either an intra- or extraperitoneal approach [3]. The guiding principle for positioning the kidney should be a smooth geometric fit in its final resting place, requiring three-dimensional visualization to ensure that the vascular and ureteral anastomoses are aligned smoothly, without tension or kinking (see Figure 3a,b).

### 3.3. Incision, Exposure, and Operative Bed Dissection

After anesthesia induction, a central venous catheter (CVC) might be inserted into the internal jugular vein for central venous pressure monitoring, optimizing fluid management, and supporting postoperative dialysis. Subclavian vein cannulation is typically avoided due to the risk of subclavian stenosis, which could hinder future vascular access if the kidney fails [9]. Similarly, an arterial catheter may also be necessary for close blood pressure monitoring or frequent blood sampling [46].

Once the patient is anesthetized, a standard 20 Ch Foley 5 mL balloon urinary catheter with a Y-connector, or a 3-way Foley catheter, should be inserted aseptically into the bladder and then connected to a drainage bag outflow and a cystoscopy tubing inflow, respectively [69]. Care is taken to avoid iatrogenic injury, especially in patients with previous prostatic or urethral issues or atrophic bladders, by ensuring proper catheter insertion techniques. The cystoscopy tubing should be connected to a liter bag containing one ampule of bacitracin–neomycin solution (1%) [69], with or without methylene blue [46]. The bladder should be rinsed once or twice with this antibiotic solution, leaving 100 mL of the solution indwelling by clamping both the inflow and outflow tubes [69]. The clamped urine drainage bag is placed beneath the head of the operating room table, allowing the anesthesiologist to manage the filling and emptying of the bladder throughout the procedure, as needed [69]. Gravity drainage is preferred for bladder distention, to prevent rupture and provide insights into the bladder capacity, aiding in the urinary reconstruction strategy assessment. This setup aids in identifying the bladder in a scarred pelvis and facilitates the execution of an extravesical UNCS [46].

Prior to the procedure, the entire abdominal area should be prepared: hair should be removed using trimmers, and the skin should be sterilized. It is essential to prepare the entire abdomen, from nipples to mid-thighs, particularly in vascular disease patients, as the incision might need to be altered or extended or may be abandoned altogether and the opposite iliac fossa opened, or saphenous vein harvesting may be required to manage a vascular reconstruction issue [5,9].

The patient is positioned supine, with the table slightly broken to hyperextend the abdomen, and rotated towards the surgeon [48]. The retroperitoneal approach to accessing the iliac arteries involves incisions through the anterolateral abdominal wall, such as the following: the oblique (modified) Gibson incision, the J-shaped “hockey-stick” lower quadrant incision, or more commonly, the oblique Rutherford Morison (or curvilinear) incision, and the Alexandre (or pararectal) incision [5,9,46,47,48,69].

The Rutherford Morison incision begins in either the right or left lower abdominal quadrant, approximately 2 cm superior to the pubic tubercle. It curves upward, paralleling the inguinal ligament, and terminates just above the anterior superior iliac spine (ASIS) of the iliac crest. During this procedure, it is crucial to avoid damaging the lateral cutaneous nerve of the thigh, which emerges through the external oblique muscle about 1 cm medial to the ASIS. For smaller adults or pediatric patients, this incision may be extended up to the costal margin to improve surgical visibility [5,9]. The external oblique muscle and its fascia are incised along the initial cut line and then dissected laterally to expand the surgical field. This incision is extended medially onto the rectus sheath, allowing for the retraction or partial division of the rectus muscle to facilitate subsequent bladder exposure. To access the peritoneum, the internal oblique and transverse muscles are cauterized along the incision line [9,69].

In contrast, the Alexandre pararectal incision takes a slightly more vertical path compared to the Rutherford Morison approach. This incision originates 2 cm above the pubic symphysis and progresses laterally and cranially along the rectus sheath’s edge, positioned two finger-widths medial to the ASIS. The junction where the oblique abdominal muscles meet the rectus sheath (known as the Spigelian fascia) is divided to reveal the underlying peritoneum [9].

Regardless which incision is used, once peritoneal exposure is achieved, a ligation and division of the inferior epigastric vessels may be performed to enhance access. However, if multiple renal arteries are present, preserving the inferior epigastric vessels initially is prudent, in case the inferior epigastric artery is needed for anastomosis to a lower polar renal artery [5,70]. Similarly, if the donor kidney presents a small, divided accessory artery or arterial branch requiring salvage during back-table preparation, the preservation or division of the superficial inferior epigastric artery is considered, maintaining a good length for potential revascularization [46,70]. Moreover, in instances where the ipsilateral superior epigastric vessels have recently been divided (e.g., during a concomitant liver transplant or recent subcostal incision for a previous ipsilateral nephrectomy, gallbladder removal, or splenectomy), preserving the inferior epigastrics is also advisable, to prevent acute rectus muscle ischemia [46]. While early descriptions of the procedure advocated for division of the spermatic cord, it is now seldom necessary for adequate exposure and should be avoided. Instead, freeing the spermatic cord laterally allows for medial retraction. In contrast, in female patients, the homologous round ligament can be confidently divided between ligatures [5,9].

**Figure 4:**
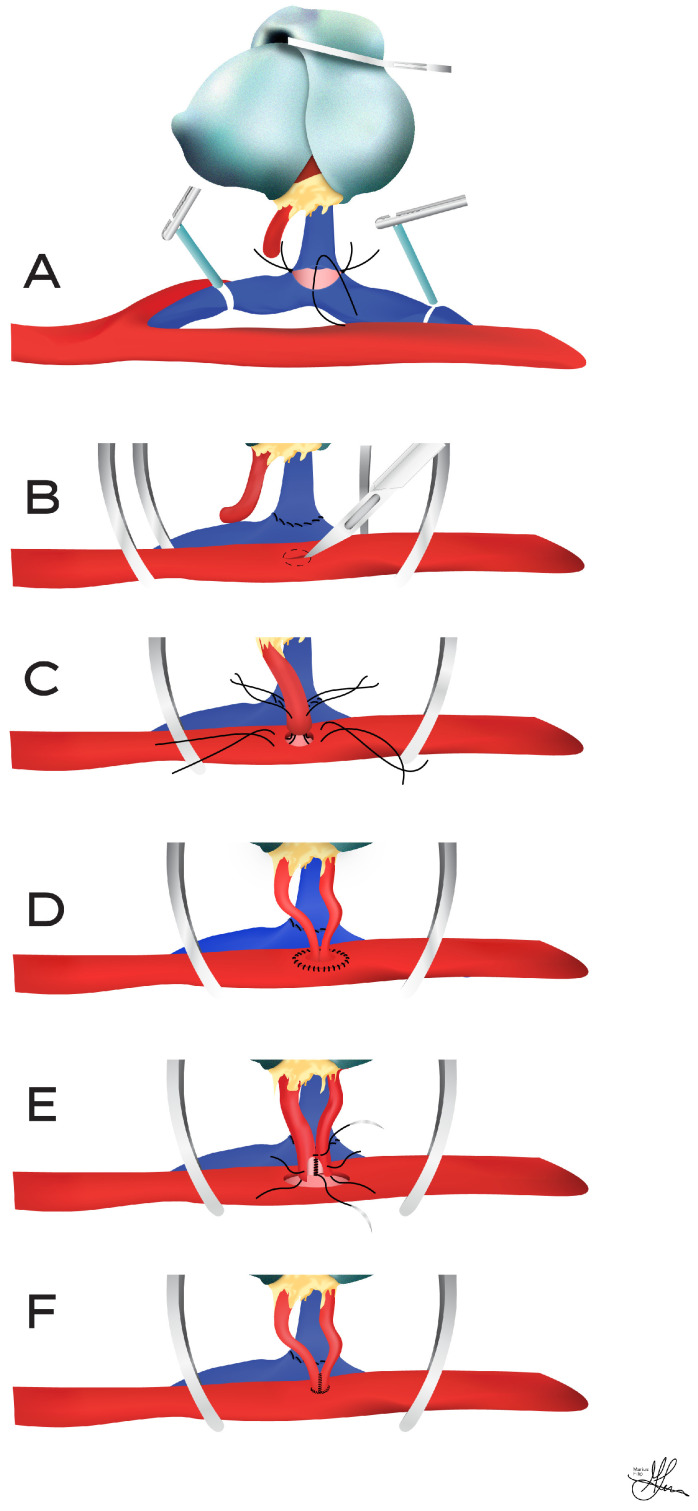
Termino-lateral vascular anastomoses using external iliac vessels: (**A**) Venous anastomosis between donor renal vein and recipient external iliac vein (quadrant technique). (**B**) External iliac artery arteriotomy (a longitudinal incision in the anterior arterial wall, with a no. 11 scalpel blade, which may be extended along the dotted line using a vascular punch instrument). (**C**) Quadrant sutures between the recipient external iliac artery and donor renal artery. (**D**) Vascular reconstruction using donor Carell patch of aorta. (**E**) Side-to-side anastomosis of donor renal arteries (pantaloons/pair of pants technique). (**F**) Vascular reconstruction after side-to-side anastomosis of donor renal arteries.

**Figure 5:**
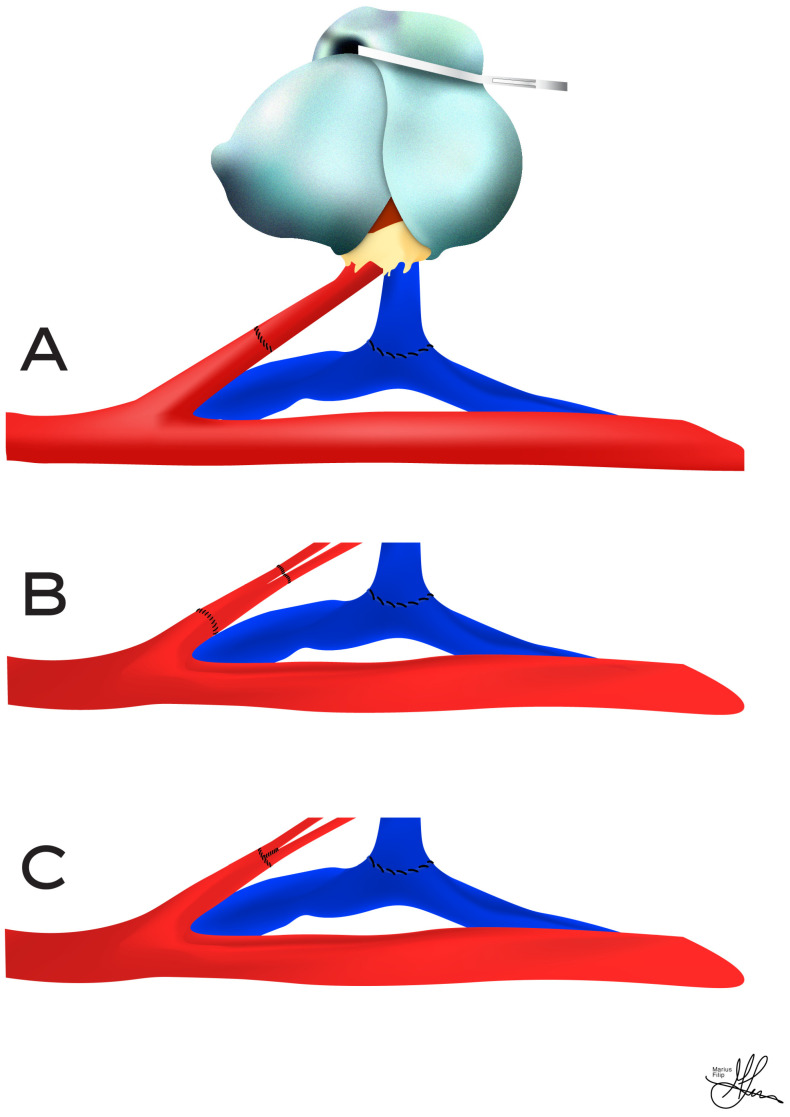
Termino-terminal arterial anastomoses using recipient internal iliac artery: (**A**) Standard vascular reconstruction using internal iliac artery (end-to-end) and external iliac vein (end-to-side)—final view; (**B**) vascular reconstruction after recipient internal iliac graft interposition; (**C**) vascular reconstruction after side-to-side anastomosis of donor renal arteries.

Onward, the procedure advances with the incision of the transversalis fascia, followed by the upward and medial reflection of the peritoneum to expose the psoas muscle and iliac vessels. This maneuver is most effectively performed in a caudal-to-cranial direction [69], detaching the parietal peritoneum from the posterior abdominal wall. This dissection continues until sufficient space is created for graft placement in the parapsoas gutter [5], and the iliac vessels are clearly visible (see Figure 3a). A self-retaining retractor is then inserted to optimize exposure and free up both of the assistant’s hands for the anastomosis [9]. The initial dissection focuses on exposing the external, common, and/or internal iliac arteries, depending on the specific circumstances. The external iliac artery is typically the preferred site (see Figure 4), particularly when a healthy Carrel patch is present on the donor renal artery (see Figure 4D). The internal iliac artery may be considered if this is not the case (see Figure 5), while the common iliac artery might be used for a second transplant or in cases of significant arterial disease affecting the external iliac vessels [9].

The lymphatics accompanying the vessels are preserved when possible and carefully separated from the artery without division. Although it has been suggested that ligating rather than cauterizing the lymphatics might prevent later lymphocele development, strong evidence supporting this practice is lacking [71]. Surgeons must exercise caution to avoid mistaking the genitofemoral nerve for a lymph vessel, as it runs along the medial edge of the psoas muscle, with a branch potentially crossing the distal external iliac artery [9,48,69]. Following the exposure of the appropriate iliac arteries, dissection of the external iliac vein begins. If a left kidney with a long renal vein is available, dissection of the external iliac vein alone generally suffices for a tension-free anastomosis [3,5,9]. Temporarily placing the cold kidney graft into the surgical site aids in selecting the optimal anastomosis sites on the recipient artery and vein (see Figure 3b).

## 4. Revascularization Techniques

Deciding which anastomosis—arterial or venous—should come first often hinges on the kidney’s final positioning and the convenience and ease of executing the subsequent anastomosis. After the graft is prepared and becomes ready for implantation, the recipient vessels should also be prepared for clamping. Some surgeons administer a modest dose of heparin (e.g., 30–60 IU/kg), while others, especially when dealing with patients already on dialysis, opt to cross-clamp the recipient vessels without heparinization [9]. Prior to making the arteriotomy or venotomy (see Figure 4B), it is crucial for the surgeon to mentally visualize the kidney in its final implantation position, considering the path the renal artery and vein will take. This visualization ensures the optimal placement of the anastomosis sites (see Figure 3b). The selection of vascular connection sites should take into account the length of the donor vessels, ensuring there is no kinking when the kidney is positioned in the iliac fossa, typically in the developed parapsoas gutter.

For arterial anastomoses, the external or common iliac arteries are generally preferred, as the internal iliac artery is more susceptible to atherosclerosis. In most instances, an end-to-side anastomosis of the donor renal artery to the recipient’s external or common iliac artery is recommended (see Figure 4), rather than an end-to-end connection to the internal iliac artery (see Figure 5). For an end-to-side anastomosis, vascular clamps are applied proximally and distally to the external iliac artery. When using the internal iliac artery, a vascular clamp is placed at its origin, or both the common and external iliac arteries are clamped. The vein is secured with vascular clamps both proximally and distally.

After dividing the internal iliac artery at its distal end, the lumen is irrigated with heparinized saline. If the external iliac artery or common iliac artery is being used, an appropriately sized arteriotomy is made, often expanded with a vascular punch (see Figure 4B), and the lumen is again flushed with heparinized saline. The venotomy undergoes a similar irrigation with heparinized saline. It is important to note that if a valve is present at the venotomy site, it must be carefully excised [3,5,9,69].

It is essential to check the inner lining of both the donor and recipient arteries before beginning the arterial connection, to ensure no damage [69]. If damage is identified, it must be repaired before or during the arterial connection. In recipients with a history of iliac or femoral vein thrombosis, pre-surgery imaging is advised to confirm the patency of at least one iliac vein and the IVC. If an unexpected thrombosis is found during surgery, the transplant may be halted [3]. With prior planning, alternate veins can be utilized for the transplant. If the patient had an iliac artery prosthetic replacement due to severe atheroma, the renal artery should connect to the prosthetic. Systemic heparin might be administered before clamping a vascular prosthesis [72].

When connecting the renal artery end-to-side to the external iliac artery (typically using a Carrel patch of the aorta—see Figure 4D), it is advised to begin with the venous anastomosis (see Figure 4A). This allows the end-to-side arterial anastomosis to become properly aligned [5]. However, if the renal artery is being attached to the internal iliac artery (see Figure 5), the arterial connection should be prioritized, as this facilitates the correct renal vein positioning [5] (see Figure 5A). Although various sutures and suturing methods exist for vascular connections, typically, 5-0 and/or 6-0 non-absorbable mono-filament polypropylene sutures are used for the renal vein and artery anastomoses [48,69] (see Figure 4A,C).

Prior to utilizing the internal iliac artery, a thorough inspection of its origin is conducted, to check for atheroma build-up. Any atheromatous disease in the common or external iliac artery should also be noted [3]. When using the internal iliac artery, it is essential to mobilize a segment of the common and external iliac arteries. This allows for lateral rotation of the internal iliac artery without kinking at its origin and facilitates the application of vascular clamps to the more accessible common/external iliac arteries, if the internal iliac is too short [5,9]. In the case of multiple graft arteries without an aortic patch (i.e., see Figure 4E), the internal iliac arterial dissection should be advanced distally, to expose its initial divisions, which may be suitable for anastomosis to individual renal arteries [9,69]. This can be done in situ, or by removing the internal iliac arterial bifurcation and performing a backbench anastomosis of the graft arteries onto the divisions of the internal iliac artery, using the resected portion of the recipient internal iliac as an interposition graft [73] (see Figure 5B). Furthermore, for living donor kidneys or other scenarios lacking a suitable aortic patch, arteries may be joined in a side-to-side (“pantaloons/pair of pants”) fashion (see Figure 4E,F and Figure 5C), or an accessory graft arterial branch may be connected to the main artery by an end-to-side anastomosis procedure. Finally, multiple graft arteries can also be restructured to a patch plasty, created from PTFE or autogenous tissue, to facilitate vascular anastomosis in the recipient [48,69].

When the graft artery is significantly longer than the vein, it can be anastomosed to the recipient internal iliac or, more simply, to the external iliac, with the graft itself fitted within a sub-rectus pouch. This pouch is created by dissecting the peritoneal lining of the posterior sheath of the rectus muscle [74], allowing the longer graft artery to run a smoother course. In rare cases involving a very short renal vein (such as with a right-sided graft, or occasionally a left-sided graft with a shortened vein), or in obese recipients, division–ligation of the internal iliac vein, and usually 1–2 gluteal veins, may be performed. This technique brings the common/external iliac veins well into the operative bed, especially if a concomitant arterial internal iliac division is performed, enabling a tension-free anastomosis [3,9]. However, this iliac/gluteal venous division maneuver carries risks, as ligation slippage may lead to hard-to-manage hemorrhage. Preferable alternatives for managing short renal veins include using the parachute technique for venous anastomosis, a more distal anastomosis placement on the external iliac vein, or employing a donor IVC segment to extend the renal vein [9]. In living renal donors, the graft vein can be lengthened using the donor gonadic vein obtained during nephrectomy [75] or with a recipient saphenous vein graft [76], although other previously mentioned methods are generally preferred.

Finally, prior to completing the arterial anastomosis, a bulldog clamp is placed on the renal artery, and the distal clamp on the external iliac artery is temporarily opened to allow bleeding, which helps flush out clots and air from the renal artery. After this, the anastomosis is finished. The clamp on the external iliac vein is then removed first, starting from proximal to distal, followed by the clamps on the external iliac artery, from distal to proximal, to restore blood flow to the kidney [5,69]. Any significant bleeding spots are identified, and additional sutures are placed as needed. The kidney is then irrigated with warm saline, and gauze swabs are used to cover the anastomosis sites.

The quality of reperfusion varies; kidneys from live donors or those that have undergone machine preservation tend to reperfuse smoothly and quickly turn firm and pink, i.e., they should start producing urine immediately [5,9,46,69]. Conversely, kidneys from deceased donors, especially those that have endured prolonged cold ischemia or were donated after circulatory death, may initially show uneven reperfusion, although this typically stabilizes over time [9].

Ensuring optimal reperfusion requires careful attention to several key factors. It is crucial to verify that all clamps have been completely removed, the artery is free from any kinking, and the recipient’s blood pressure is maintained at an appropriate level. Additionally, surgeons must carefully inspect both the proximal recipient artery and the graft artery for signs of intimal dissection, which can occur due to traction during donor organ retrieval or severe hypertension in the donor during harvesting. In cases where concerns about reperfusion quality persist, the Hume test can provide valuable reassurance. This diagnostic maneuver involves temporarily occluding the renal vein using finger and thumb pressure, which causes the kidney to swell and pulsate. Upon releasing the vein, a palpable softening of the kidney occurs as its turgor diminishes. This simple yet effective test helps confirm adequate arterial inflow and venous outflow, offering important insights into the graft’s initial vascular functionality [9,69].

## 5. Urinary Tract Reconstruction Techniques

**Figure 6:**
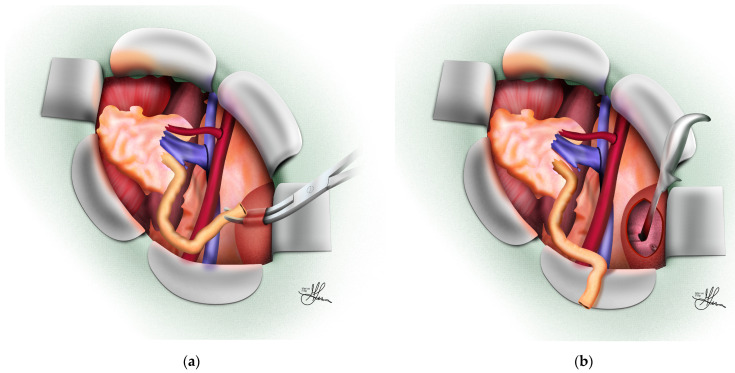
Ureteroneocystostomy techniques for urinary reconstruction in renal transplantation: (**a**) Extravesical Lich–Gregoir; (**b**) transvesical Leadbetter–Politano.

Once a satisfactory reperfusion of the renal allograft is achieved and confirmed, the focus shifts towards urinary drainage, as we enter the urinary tract reconstruction phase of the surgery. The chosen reconstruction method hinges on the length and state of the donor’s ureter, as well as the recipient’s ureter/bladder. Typically, a Lich–Gregoir extravesical UNCS technique (or one of its variations) is employed (see Figure 6a), as it offers significant advantages over the classic Leadbetter–Politano transvesical method (see Figure 6b), such as no need for an additional cystotomy and less donor ureteral length requirement. This approach tends to be associated with reduced operative times and lower rates of ureteral complications, such as ischemia, obstruction, hematuria, and urine leaks [5,26,77,78]. The transplant ureter is typically routed behind the spermatic cord to avoid potential kinking and obstruction [5]. Generally, this is straightforward; however, dividing the spermatic cord can sometimes facilitate the UNCS without significant long-term consequences [5,9,48].

The graft ureteral stump will be tailored to the needed length, then the distal end will be further spatulated, in order to facilitate a tension-free anastomosis to the vesical wall, ensuring good perfusion at its distal end to minimize the risk of ischemia, which could additionally lead to ureteral strictures or leaks [46]. To prepare the bladder for anastomosis, it must be distended with ~150–200 mL of saline, which will help in dissecting the individual bladder wall layers [69]. To further aid in clearly identifying the bladder during cystotomy, especially in obese or peritoneal dialysis patients, in which the peritoneum might be mistaken for the bladder, a methylene blue-tinted irrigation can be instated instead via the same Foley catheter inserted preoperatively, and may prove to be invaluable [46,48,69].

In the Lich–Gregoir technique, as seen in Figure 6a, the sero-muscular layer of the superior lateral wall of the distended bladder is then incised, either continuously, ~2.5–3 cm in the direction of the ureter [22], or in an interrupted manner, with 2 small parallel incisions, perpendicular to the ureter, causing the mucosa to bulge at the incision site [54]. A plane is established between the mucosa and the muscular wall, on both sides of the incisions, extending roughly 5 mm, to later form the submucosal tunnel. An opening, approximately 1-cm-wide, is made at the distal end of the bulging mucosa [69,79].

The toe end of the ureter is anchored to the full thickness of the vesical wall [79]. The ureter-to-bladder mucosa-to-mucosa anastomosis, which involves the full thickness of the ureteric wall and the mucosa of the bladder, is typically sewn using a fine, slowly absorbable, monofilament suture (often 5-0 Polydioxanone—PDS), in a continuous running fashion [46,79]. Herein, the absorbable nature of the sutures used is of paramount importance, as this practice aims to avoid the persistence of intravesical foreign bodies (i.e., precipitation nuclei), which could subsequently lead to urinary calculi development and tract infections [46]. A JJ ureteral stent (5 Ch diameter, 12 cm in length [79]) may be used at the surgeon’s discretion, either routinely or more selectively, i.e., if concerns about the integrity of the anastomosis arise prior to its completion [9]. Although not universally endorsed, mainly due to reflux and associated infectious concerns [46], routine prophylactic stenting has been shown to significantly reduce urologic complications in RT with extravesical UNCS, according to a recent meta-analysis [80].

Lastly, the bladder’s detrusor muscle will usually be reapproximated over the finalized urinary anastomosis, using 2-0 or 3-0 Polyglactin (Vicryl) absorbable interrupted sutures [79], effectively creating a submucosal tunnel for the ureter, both for the protection and stabilization of the anastomosis, but also to serve as a potential anti-reflux mechanism and to alleviate tension accumulation on the anastomosis when the bladder is distended/full [46,47]. However, during this re-approximation, care must be taken to avoid overly tightening the resulting submucosal tunnel, as doing so may cause uropathic obstruction and early graft failure [5,9,69,79].

From a strategic standpoint, these UNCS techniques allow for the subsequent further use of native donor ureter(s), should the need for further urinary drainage revisions arise, in the context of post-RT ureteral complications. Moreover, UNCS might be preferred if the recipient’s bladder is atrophic or otherwise unsuitable. If the donor ureter is ischemic or unsuitable, a pyeloureterostomy may be considered. Importantly, during any mobilization of the native ureter for anastomosis, maintaining an adequate blood supply, by the delicate manipulation and preservation of peri-ureteral fatty tissues, is crucial to decrease the likelihood of future urologic complication occurrence. Even so, anastomoses involving the native ureter are typically technically challenging and should be performed over an indwelling JJ catheter [5,9,46].

When faced with both donor and recipient ureteral unsuitability, a pyelovesicostomy could be an option, albeit usually requiring a variable degree of bladder mobilization. Herein, techniques such as the psoas hitch or Boari flap may be necessary to enable the tension-free approximation of the recipient bladder to a short donor ureter or directly to the donor renal pelvis. In donor kidneys featuring complete ureteral duplicity, the two individual ureteral stumps will generally undergo separate UNCS procedures, unless these ureteral stumps run too close to each other, in which case the medial walls of both ureters are sewn together after spatulation, before performing a single anastomosis between this new common donor ureteral trunk and the recipient bladder wall [5].

Lastly, in recipients with a prior history of vesical augmentation or with a urinary intestinal conduit, careful tactical planning is paramount to achieving adequate urinary drainage post-RT, taking into account the appropriate recipient structure for the graft ureter implantation, the vascular supply of the preexisting augmentation or conduit, and the positioning of the kidney. Typically, these more complex urinary reconstructive procedures are performed over a protective indwelling ureteral stent [46].

## 6. Wound Closure and Postoperative Management

Closing the abdominal wall, though often given less emphasis, is a critical component of the operation that can influence long-term outcomes. Careful graft positioning is vital to prevent vessel or ureteral twisting, kinking, or obstruction. When limited extraperitoneal space raises concerns about allograft perfusion after fascial closure, the peritoneum may be opened widely to accommodate the kidney intraperitoneally [5]. This adaptive approach ensures optimal graft placement and function, addressing individual anatomical challenges. Wound closure techniques may vary, but will commonly involve using loop nylon for abdominal wall reconstruction, in two layers: the internal oblique and transverse muscles closed en mass together as a first layer, followed by the external oblique muscle approximation. The skin is typically closed with subcuticular nylon or polyglactin sutures [69,79].

The use of drains remains a debated issue, due to the potential risk they pose as a portal of entry for infectious microorganisms. If drains are deemed necessary, i.e., in cases with heightened risks of peri-graft collections of blood, lymph, or urine, a closed suction drainage system should be employed, and these drains should be removed as soon as it is feasible to reduce the risk of infection [5,48,70], i.e., once the output decreases to an acceptable level. If the drain output remains high and is clear/pale-yellow colored, it should be analyzed biochemically, at least for creatinine, to assess for a potential urine leak.

Postoperatively, the Foley catheter is generally left in place for 2–4 days to help decompress the bladder. In situations when facing a challenging/fragile urinary anastomosis, or where vesical non-compliancy might subject the new anastomosis to excessive voiding pressures, the catheter might be maintained even longer. Should there be concerns about potential urinary leakage, a retrograde cystography will be useful to check for contrast extravasation before catheter removal. The ureteral stent, if attached to the indwelling catheter, is usually removed early on postoperatively as well, or otherwise cystoscopically, within the first few months post-RT [46].

Technical complications have significantly decreased over the years due to improvements in surgical techniques, making them less common compared to liver or pancreas transplants. However, given advancements in immunosuppressive therapy in reducing renal transplant failure rates caused by acute or chronic rejection, surgical complications still represent an important source of graft failure post-RT [81].

Early postoperative issues include potential bleeding, indicated by the classic clinical signs of hemorrhagic shock, such as tachycardia, hypotension, and oligoanuria, alongside excessive pain, tenderness, distension, or a palpable mass at the surgical site. Lab work might show a drop in hemoglobin/hematocrit, but a (hyper)acute massive hemorrhage may not always be apparent biologically. Thus, postoperative hemorrhage remains largely a clinical diagnosis and should be suspected in any post-RT case showing hemodynamic instability. Possible bleeding sources include the vascular anastomoses, the allograft itself, and/or the recipient operative field. Although bleeding is often self-limiting and not detrimental to graft function, significant or persistent bleeding may necessitate surgical revision. Reintervention not only allows for the evacuation of any on-site hematoma, thus relieving the compression of the allograft and surrounding structures, but also provides an opportunity to inspect the allograft and its anastomoses and obtain a biopsy if needed. Generally, re-exploration is preferred over allowing for exsanguination or the complications of a large, undrained hematoma [46,69].

The sudden onset of oliguria/anuria, especially immediately post-RT, demands careful evaluation of the vascular reconstruction integrity to rule out allograft thrombosis and should prompt a review of the urinary drainage system, to address any common obstructive mechanical issues, namely, catheter occlusion from kinks, displacement, or clots. Patients with catheter issues often report bladder fullness and require prompt catheter patency assessment and, if need be, reestablishment. Hematuria, though typically self-limiting, i.e., resolving spontaneously, without the need for further reinterventions, can still complicate the postoperative course, especially if clots begin to form, obstructing urine outflow and thus mechanically stressing the newly achieved urinary anastomosis. Clots can usually be managed by gentle catheter irrigation with sterile water or saline. However, in some cases, replacing or upsizing the urinary catheter might be necessary. Aggressive interventions such as cystoscopy, manual vesical irrigation, or unsupervised continuous vesical irrigation should be avoided early post-RT to prevent infections and further disruption of the UNCS, and/or bladder rupture [23,46].

## 7. Conclusions

This pictorial essay provides a comprehensive overview of the surgical strategies and specific individual techniques involved in RT, focusing on the practical nuances and clinical challenges of this life-saving procedure. Herein, we provide a detailed visual exploration of the intricate anatomy and surgical processes necessary for both renal allograft retrieval from the donor and also for an adequate implantation in the recipient. Overall, we emphasize the pivotal role of careful donor and recipient selection, meticulous surgical execution, and rigorous postoperative management in optimizing patient outcomes. Innovative technologies and surgical practices that have already significantly improved the safety and effectiveness of RT stand testament to the importance of further scientific inquiry, conceptual developments, and clinical integration. Furthermore, it underscores the necessity of an interdisciplinary team approach in navigating the complexities of RT. Moving forward, it is essential that the medical community continues to refine these strategies and advocate for equitable access to transplantation, ensuring that advancements in the field translate into real-world benefits for all patients grappling with ESRD.

## Data Availability

The data presented in this study are available on request from the corresponding author.
